# Generating Tooth Organoids Using Defined Bioorthogonally Cross-Linked Hydrogels

**DOI:** 10.1021/acsmacrolett.4c00520

**Published:** 2024-11-12

**Authors:** Xuechen Zhang, Nicola Contessi Negrini, Rita Correia, Paul T. Sharpe, Adam D. Celiz, Ana Angelova Volponi

**Affiliations:** † Centre for Craniofacial and Regenerative Biology, Faculty of Dentistry, Oral & Craniofacial Sciences, King’s College London, Guy’s Hospital, SE1 9RT London, U.K.; ‡ Department of Bioengineering, Imperial College London, W12 0BZ London, U.K.; § The Francis Crick Institute, NW1 1AT London, U.K.

## Abstract

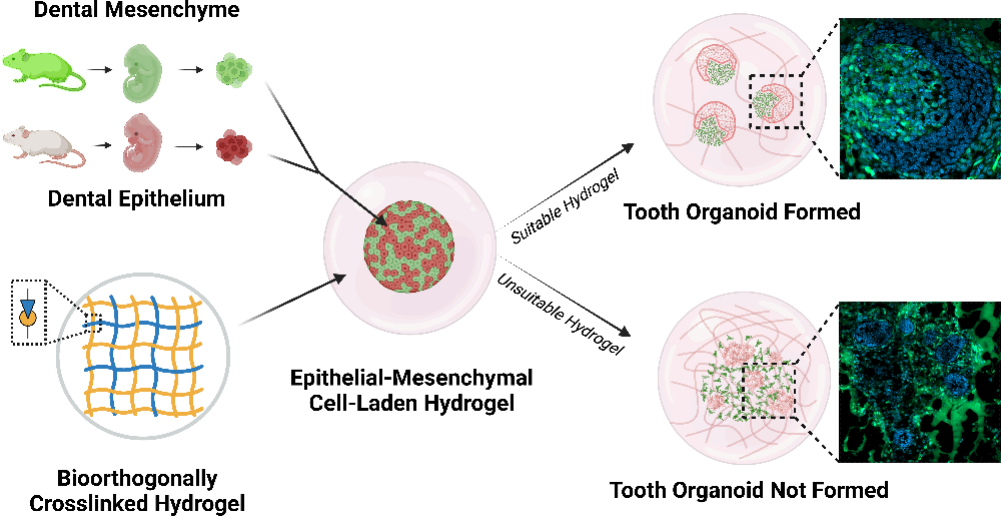

Generating teeth *in vitro* requires mimicking tooth developmental processes. Biomaterials are essential to support 3D tooth organoid formation, but their properties must be finely tuned to achieve the required biomimicry for tooth development. For the first time, we used bioorthogonally cross-linked hydrogels as defined 3D matrixes for tooth developmental engineering, and we highlighted how their properties play a pivotal role in enabling 3D tooth organoid formation *in vitro*. We prepared hydrogels by mixing gelatin precursors modified either with tetrazine (Tz) or norbornene (Nb) moieties. We tuned the hydrogel properties (*E* = 2–7 kPa; *G*′ = 500–1500 Pa) by varying the gelatin concentration (8% vs 12% w/V) and stoichiometric ratio (Tz:Nb = 1 vs 0.5). We encapsulated dental epithelial-mesenchymal cell pellets in a library of hydrogels and identified a hydrogel formulation that enabled successful growth kinetics and morphogenesis of tooth germs, introducing a defined tunable platform for tooth organoid engineering and modeling.

Tooth loss is a prevalent oral health issue affecting millions of individuals worldwide, caused by different factors like dental caries, periodontal disease, dental trauma, and some systematic diseases.^1^ Beyond impairing chewing and speaking, tooth loss also causes aesthetic and psychological issues. Current replacement solutions, including removable and fixed dentures and dental implants, are nonbiological and often fail to fully restore natural tooth form and function. Consequently, research has focused on biological tooth replacement strategies, with tissue engineering emerging as a promising approach. This method leverages cells, biomaterials, and growth factors to engineer tooth structures that mimic the features and functions of natural teeth.^2^

The goal of regenerative dentistry is to bioengineer an entire tooth, which requires replicating the interactions between the dental epithelium and mesenchyme. In 1987, Mina and Kollar found that early first branchial arch epithelium can stimulate nonodontogenic, neural-crest-derived mesenchymal cells in the second arch to form dental papilla.^3^ When dissociated dental epithelial and mesenchymal cells derived from mice embryos are reassociated *in vitro*, tooth germ-like structures were formed.^4,5^ These tissue-engineered cell-based *in vitro* structures were shown to recapitulate the developmental aspects of the complex structures of the corresponding *in vivo* dental tissues and develop into a functional organ^4^ and, therefore, can be defined as organoids.^6^

Organoids are formed from embryonic and adult pluripotent or tissue-resident stem cells, as well as progenitor or differentiated cells derived from healthy or diseased tissues.^6,7^ Tooth organoids are defined as three-dimensional (3D) *in vitro* structures that replicate the developmental processes and structural complexity of natural teeth. These organoids are derived from dental cells, which can be of either embryonic or adult origin. The development of tooth organoids involves the self-organization of cells into structures that mimic the cellular composition and functional attributes of the actual teeth. When these structures are transplanted *in vivo*, they can then fully develop into mature teeth.^8,9^ While these studies used tissues and cells derived from embryonic origin, Ohazama et al. used adult bone marrow-derived mesenchyme cells and embryonic early, inductive, dental epithelium resulting in tooth formation *in vitro*, demonstrating that adult mesenchymal cells can actively participate in tooth development.^10^ Similarly, our previous research showed that human gingival epithelial cells respond to embryonic, mouse mesenchyme signals, leading to the formation of teeth.^11^ These studies demonstrate that bioengineered organ replacements hold promise as a regenerative therapy. To achieve fully functional bioengineered teeth, cells from adequate sources should be gathered in suitable 3D artificial matrixes (biomaterials) that must support cell self-organization and promote appropriate tooth morphogenesis.^12^

Different biomaterials have been investigated to support mesenchymal-epithelial crosstalk to regenerate teeth, including polyglycolide acid (PGA), poly-l-lactide acid (PLLA), and poly(lactic-*co*-glycolic acid) (PLGA),^13−16^ collagen sponges,^17^ and decellularized tooth buds.^18^ Cell-laden hydrogels have been also tested to provide a 3D environment supporting cell encapsulation, growth and rearrangement,^19^ including collagen^20,21^ and Matrigel.^22,23^ Despite these biomaterials serving as successful 3D matrixes for cell crosstalk and tooth bud (tooth germ-like structures) formation, the lack of information and limited control and tunability of their physicomechanical properties hinder our capability of fully understanding the pivotal role that biomaterials play in tooth organoid formation *in vitro*.^24,25^

In this work, we engineered cell-laden bioorthogonally cross-linked gelatin hydrogels and investigated for the first time their potential for tooth primordia engineering. Compared to other biomaterials, these hydrogels allow fine-tuning of their properties by varying different design parameters, enabling a better understanding on the involvement of biomaterial properties in determining tooth growth kinetics and morphogenesis *in vitro*. We cross-linked gelatin using the inverse-electron demand Diels–Alder reaction between tetrazine (Tz) and norbornene (Nb) to obtain cell-laden gelatin-based bioorthogonally cross-linked click hydrogels.^26^ We tuned the physicomechanical properties of the hydrogels by modifying the biomaterial design (i.e., concentration and ratio between Tz and Nb) to obtained controlled and defined 3D matrixes for cell culture, and we investigate their potential in supporting odontogenic interactions between dental epithelial cells and mesenchymal cells to generate tooth organoids (Figure 1).

**Figure 1:**
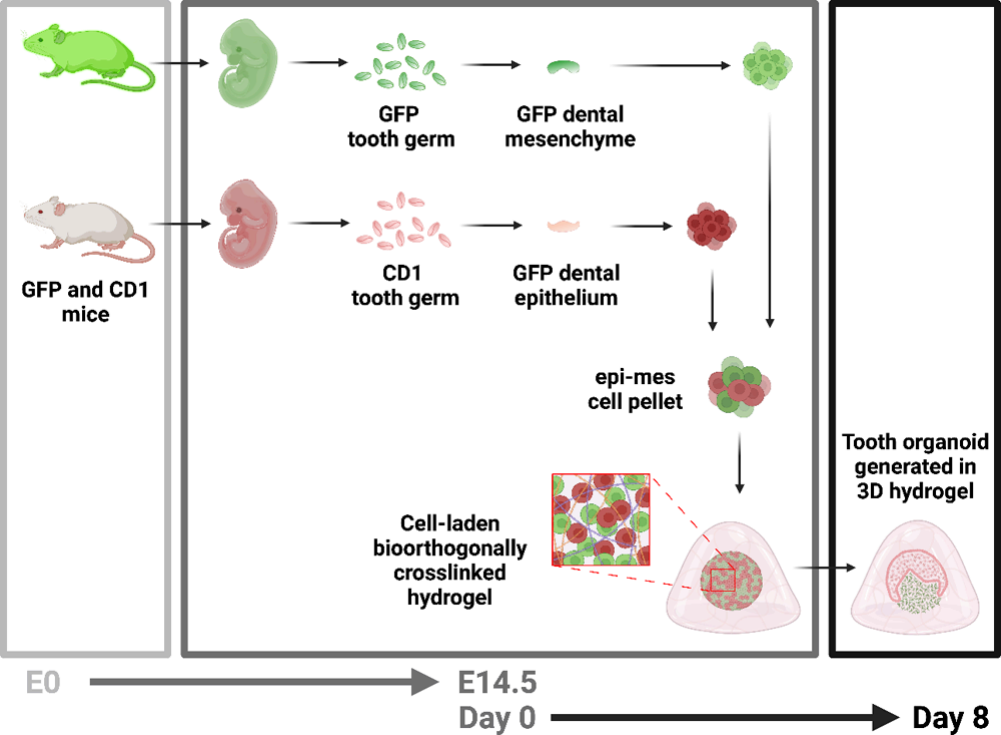
Preparation of the 3D tooth organoids. Dental mesenchyme and epithelium were obtained in green fluorescent protein (GFP) and CD1 mouse embryos at embryonic day 14.5 (E14.5), separately. After digesting to single cell suspensions, the two cell populations were combined to obtain an epithelial-mesenchymal cell pellet, encapsulated in hydrogels (day 0), and cultured *in vitro* to generate 3D tooth organoids (day 8). Created with BioRender.com.

We chemically modified gelatin with either tetrazine or norbornene moieties (GEL_Tz or GEL_Nb) to obtain the hydrogel precursors, and we verified and quantified the polymer functionalization via ^1^H NMR. After polymer functionalization, we identified the characteristic peaks of Tz and Nb within the ^1^H NMR spectra of the functionalized gelatin, compared to nonfunctionalized gelatin (Figure 2B). The DOM for both GEL_Tz and GEL_Nb was 0.10 ± 0.01 mmol g^–1^ of gelatin.

**Figure 2:**
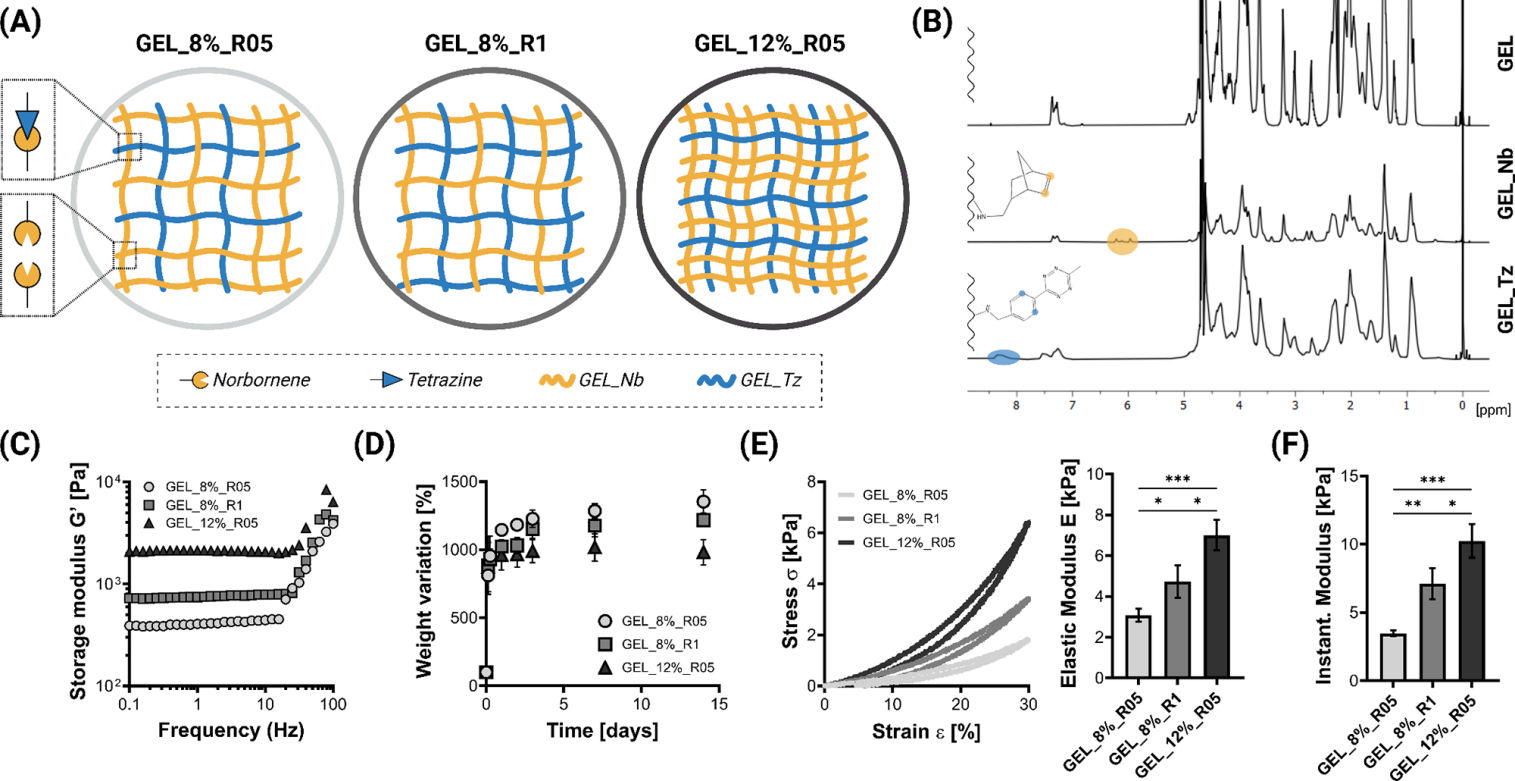
Bioorthogonally cross-linked click gelatin hydrogels. (A) Schematic of hydrogel structures prepared by varying the concentration (8% and 12% w/V) and ratio between gelatin modified with tetrazine (GEL_Tz, blue lines) and gelatin modified with norbornene (GEL_Nb, yellow lines) at 1:1 (R1) or 0.5:1 (R05). (B) Representative ^1^H NMR spectra of unmodified gelatin (GEL), gelatin modified with norbornene (GEL_Nb), and gelatin modified with tetrazine (GEL_Tz). (C) Representative rheological curves of the frequency response of the cross-linked hydrogels. (D) Swelling and weight variation in culture medium of the cross-linked hydrogels (*n* = 4). (E) Representative stress–strain curves (σ–ε) of cross-linked swollen hydrogels and elastic modulus (*E*). (F) Instantaneous modulus calculated from indentation tests (*n* = 3; * *p* < 0.05, ** *p* < 0.01, *** *p* < 0.001). Created with Biorender.com.

We then prepared hydrogels by mixing the GEL_Tz and GEL_Nb precursors (Figure 2A, Table S1), enabling the bioorthogonal click reaction between tetrazine and norbornene. All hydrogels successfully cross-linked after mixing the hydrogel precursors. We then evaluated the hydrogel rheological properties after cross-linking. Rheological tests confirmed the hydrogel formation and evidenced a higher storage modulus *G*′ for hydrogels prepared with a higher polymer concentration and stoichiometric ratio (Figure 2C). The estimated mesh sizes ξ were 27, 22, and 17 nm for GEL_8%_R05, GEL_8%_R1, and GEL_12%_R05, respectively. We performed swelling tests to evaluate the stability of the cross-linked hydrogels in *in vitro* cell culture-like conditions. All hydrogels were stable in culture medium at 37 °C for at least 2 weeks, showing the possibility of using these hydrogels as platforms for cell cultures, and different swelling profiles were identified (Figure 2D): at plateau, GEL_8%_R05 was more swollen than GEL_8%_R1, which was in turn more swollen than GEL_12%_R05. We then investigated the mechanical properties of the hydrogels and the possibility of tuning them by varying the hydrogel design. The swollen hydrogels were characterized by different compressive mechanical properties (Figure 2E, left). All hydrogels showed a hysteresis cycle during the compression test, characterized by an increase in stress during loading and a decrease in stress during unloading. The mechanical properties were tuned by varying the hydrogel concentration and the stoichiometric ratio. The elastic moduli *E* varied between 2 and 7 kPa approximatively (*E*_GEL_8%_R05_ < *E*_GEL_8%_R1_ < *E*_GEL_12%_R05_; Figure 2E, right). A decrease in *E* during swelling was observed (Figure S1) and attributed to the absorption of aqueous medium and decrease in polymer density in the 3D hydrogel network (Figure S2). Similarly, the Instantaneous Modulus measured by indentation tests increased by increasing the polymer concentration and by using a 1:1 Tz:Nb ratio (Figure 2F). All hydrogels were successfully used to incorporate viable cells in the biomaterial 3D structure (Figure S3A); after 1 day of culture, the percentage cell viability was >70% for all the tested hydrogels (Figure S3B), confirming their cytocompatibility. These results show the possibility of tuning the hydrogel physicomechanical properties by varying different design parameters, including the hydrogel concentration and the ratio between the hydrogel precursors, to obtain cytocompatible defined hydrogels.

By injecting mouse embryo whole tooth germs at embryonic day 14.5 (E14.5) inside the three different hydrogel formulations (Figure 3**A1–3**), we assessed the influence of the hydrogel physicomechanical properties on the growth kinetics and morphological development of tooth germs. After comparing with the physiological tooth growth *in vivo* (Figure S4), we found that increased hydrogel rheological and mechanical properties decreased tooth germ growth rates, indicating that the hydrogel matrix biomechanically regulates tooth germ development. After 8 days of *in vitro* culture, tooth germ morphology in the GEL_8%_R1 group (Figure 3**A2** and Figure 3**C2**) and GEL_12%_R05 group (Figure 3**A3** and Figure 3**C3**) exhibited absence of characteristic late cap stage structures with typical inner and outer enamel epithelium and stellate reticulum (Figure S4G), as well as more condensed mesenchymal cells, in contrast to the more defined and structured tooth germs in the GEL_8%_R05 group (Figure 3**A1** and Figure 3**C1**). Tooth germs encapsulated in lower stiffness hydrogels displayed typical structural development, displaying an observable inner and outer enamel epithelium and stellate reticulum (Figure S4G).

**Figure 3:**
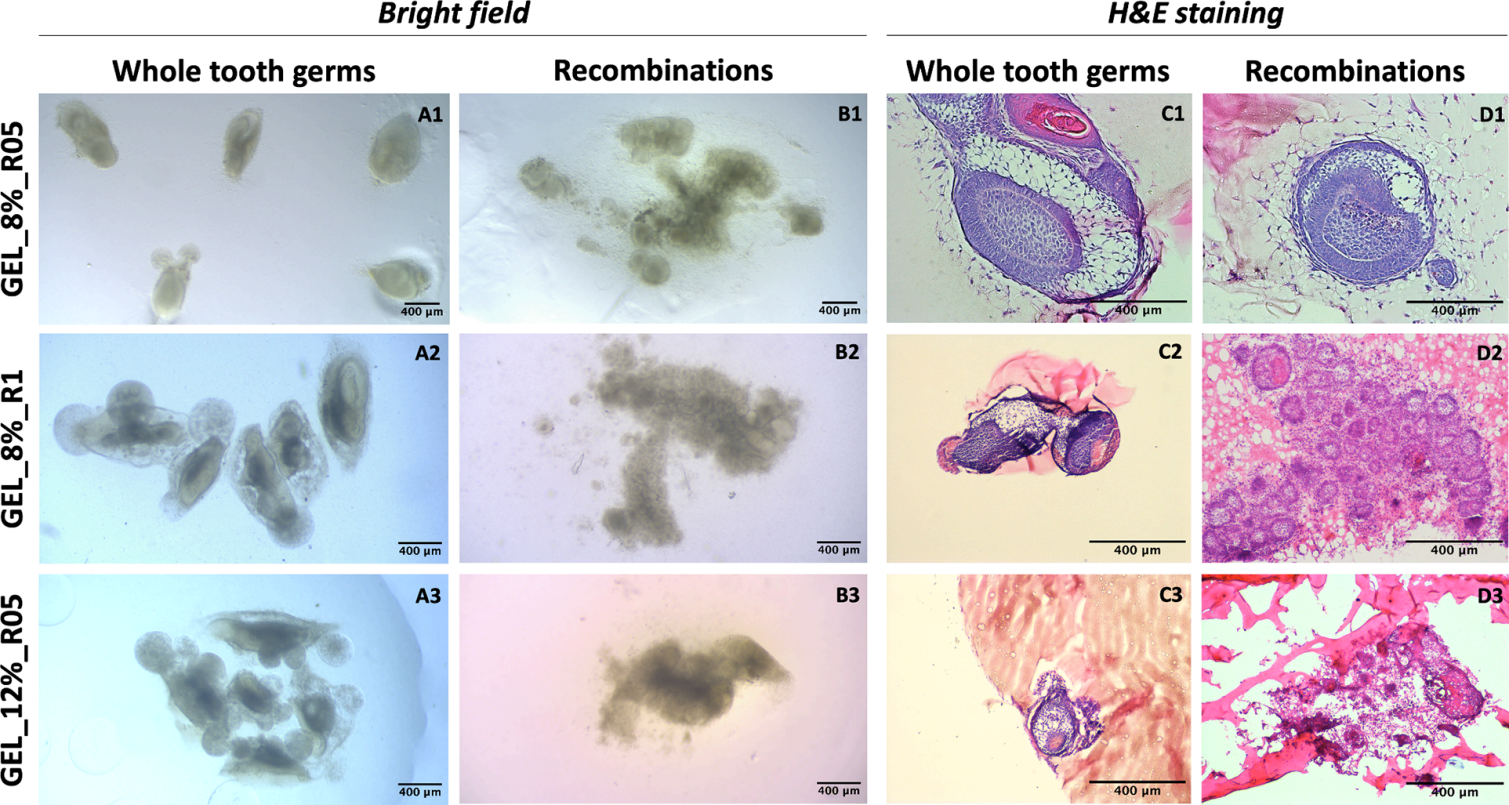
Whole tooth germs and recombination of dental mesenchymal and epithelial cells in bioorthogonally cross-linked hydrogels prepared by varying polymer concentrations and stoichiometric ratios. **(A1–3)** Tooth germs in GEL_8%_R05 (*n* = 6), GEL_8%_R1 (*n* = 6), or GEL_12%_R05 (*n* = 6). **(B1–3)** Recombination in GEL_8%_R05 (*n* = 13), GEL_8%_R1 (*n* = 9), or GEL_12%_R05 (*n* = 6). **(C1–3)** H&E staining images for **A1–3**. **(D1–3)** H&E staining images for **B1–3**. Scale bar: 400 μm.

Observed morphological differences demonstrated that hydrogel properties influence the structural progression of encapsulated tooth germs. As the ratios between the GEL_Tz and GEL_Nb precursors was changed from 0.5 to 1, a higher number of chemical cross-links formed while keeping the gelatin concentration constant (GEL_8%_R05 vs GEL_8%_R1), the hydrogel rheological and mechanical properties increased, resulting in decelerated growth kinetics, accompanied by changes in morphology. Similarly, increasing the hydrogel concentration while maintaining the GEL_Tz and GEL_Nb ratio constant (GEL_8%_R05 vs GEL_12%_R05) resulted in slower tooth growth and morphogenesis.

We also investigated the impact of the hydrogel design on tooth organoid formation in an *in vitro* model. The formation of tooth organoids was observed exclusively in the GEL_8%_R05 group. The consistent formation of tooth organoids in this group (*n* = 13), suggests that the hydrogels with lower mechanical and rheological properties facilitate the tooth organoid formation (Table S2). In contrast, tooth organoids formed only once in the GEL_8%_R1 group (*n* = 9). In the GEL_12%_R05 group, the stiffest of the three hydrogels tested, tooth organoids did not form (*n* = 6), suggesting that hydrogels with increased stiffness are less favorable in the formation of dental organoids.

Brightfield microscopy did not discern detailed morphological differences across the hydrogel samples. However, compared to the images taken at day 0 (Figure 1 and Figure S5), which show cells instead of structures concentrated inside the hydrogel, images captured after 8 days of culture (Figure 3**B1–3**) reveal visible round structures indicating self-sorting and cellular crosstalk in each group. To reveal the structural characteristics of formed organoids, histological analysis of tissue sections stained with H&E had been performed. In the GEL_8%_R05 group, histological analysis revealed the presence of well-defined tooth organoid with an epithelium, and condensed mesenchyme (Figure 3**D1**), which was similar to the entire tooth germs group in the same hydrogel ratio (Figure 3**C1**), indicating a successful development of tooth organoids within this hydrogel environment. In contrast, we found epithelial cysts and histologically undefined, round structures in the other groups (Figure 3**D2–3**). Based on these histological differences, hydrogels of higher stiffness showed a decreased ability to enable organoid formation.

Immunofluorescence was used to determine the cellular composition and to clarify the origin and relationship between epithelial and mesenchymal cells within the tooth organoids. We found that tooth organoids in GEL_8%_R05 hydrogel contained epithelial and mesenchymal cells recombined from different origins (Figure 4**A1-A6;**Video S1, Video S2). After staining with DAPI, both epithelial and mesenchymal cells had been stained and showed blue fluorescence, while only mesenchymal cells derived from GFP mice showed green fluorescence. This can provide evidence that the tooth organoid that formed in the hydrogel is a new-form structure. The dynamic interplay of epithelial and mesenchymal cells in the GEL_8%_R05 hydrogel environment indicated dental organoid formation. The other two hydrogel formulations did not support tooth organoid formation, as evidenced by the absence of organogenesis via fluorescence staining (Figure 4**B1–6** and Figure 4**C1–6**). Specifically, mesenchymal cells (GFP^+^) were located outside of the round structures which formed by only epithelial cells (GFP^–^), indicating they had little or no communication with epithelial cells. These results suggest that tuning the hydrogel properties is pivotal in modulating the occurrence of tooth organoids, providing valuable insight for tissue engineering and regenerative dentistry applications in the future.

**Figure 4:**
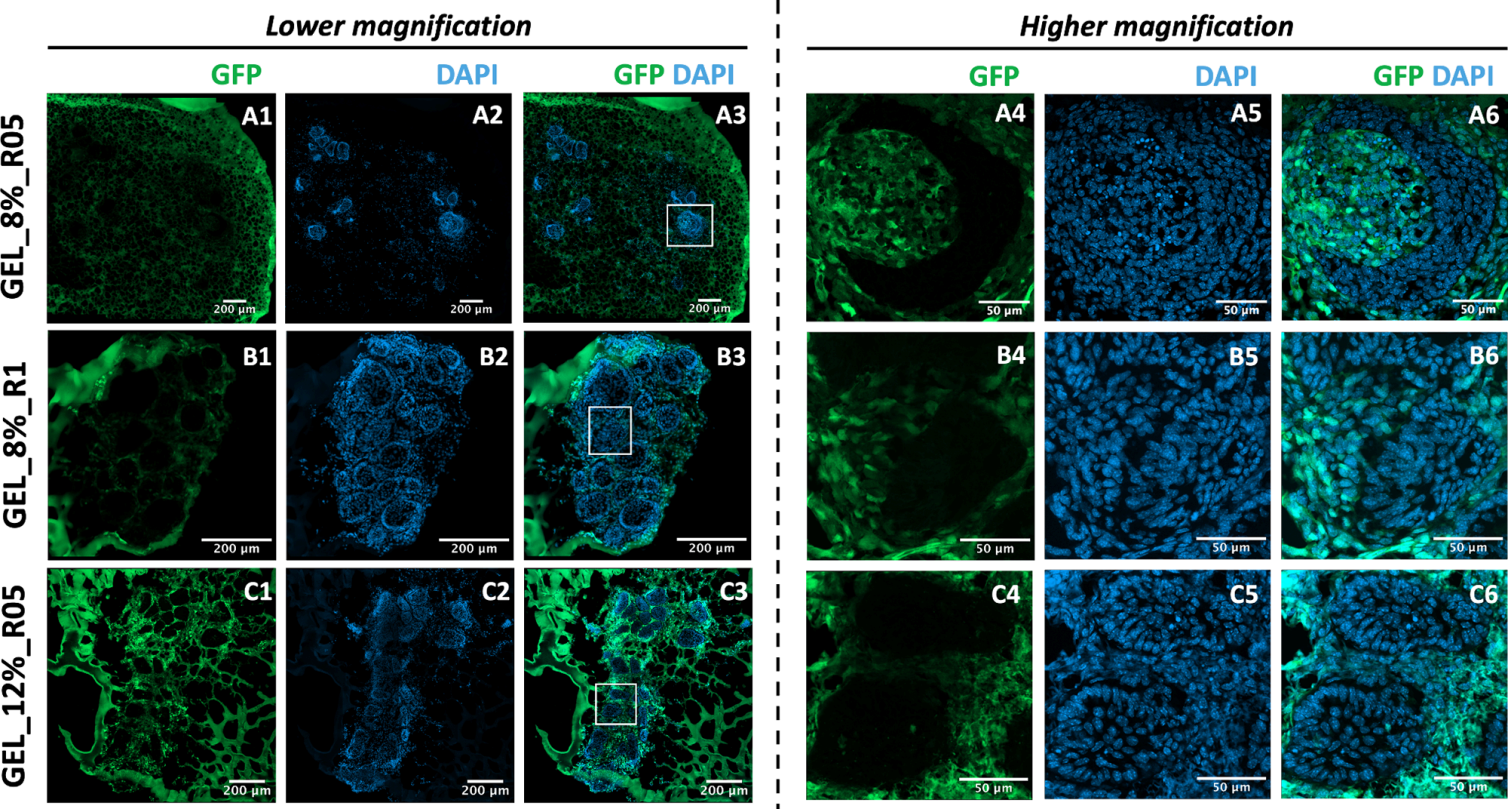
Recombination of GFP dental mesenchymal cells and CD1 dental epithelial cells in bioorthogonally cross-linked hydrogels prepared by varying polymer concentrations and stoichiometric ratios. (A) GEL_8%_R05, (B) GEL_8%_R1, and (C) GEL_12%_R05 (1–3 are in low magnification; 4–6 are the small white squares in A3, B3 and C3 in high magnification.) Scale bar: 200 μm in lower magnification and 50 μm in higher magnification.

Reproducing the biological mechanisms of embryogenesis *in vitro* is essential to engineer developmental tissue engineering strategies for tooth repair and *in vitro* platform for dental development pathophysiological studies.^12,27^ The interaction between the oral epithelium and mesenchyme initiates tooth development, forming tooth buds that progress through bud, cap, and bell stages (Figure S4). These stages lead to the development of the enamel organ, dental papilla, and dental follicle, ultimately forming dental tissues such as enamel, dentin, and supporting structures.^28^ To regenerate teeth through tissue engineering, epithelial and mesenchymal cells must interact within a scaffold that facilitates these interactions, resulting in tooth organoids that can fully develop into teeth *in vivo*.^4^

Producing artificial extracellular matrix (ECM) for tissue engineering and *in vitro* modeling requires synthesizing cytocompatible hydrogels with customizable and defined characteristics. The choice of polymer and its cross-linking mechanism are crucial in regulating the physical and mechanical characteristics of the hydrogel. This is essential to attain biomimetic and cytocompatible hydrogels that can effectively guide the desired cellular communication.^24^ Here, we examined gelatin-based hydrogels obtained by the bioorthogonal click reaction between Tz and Nb to mimic ECM and support tooth organoids’ formation.

We selected gelatin as the polymer to form the hydrogel networks. As a collagen derivative, gelatin is characterized by several advantages including versatility of fabrication, availability, presence of cell-adhesive motifs (i.e., RGD sequences that promote integrin-mediated interactions), biodegradability, and lower immunogenicity and antigenicity compared to collagen.^29^ Moreover, gelatin hydrogel properties can be tuned by varying several design parameters including gelatin source, polymer concentration, cross-linking methods, and cross-linking density.^30^ We then selected the click reaction between Tz and Nb to prepare our cell-laden hydrogels due to its bioorthogonal nature, enabling the cross-linking reaction to occur in physiological conditions with no interference with native biochemical processes,^31^ and the tunability of the material properties prepared using this cross-linking chemistry.^32^ Moreover, this hydrogel formulation can be simply prepared by mixing hydrogel precursors to allow their spontaneous cross-linking. We then altered the biomaterial design parameters to tune the properties of the hydrogels. In our previous study, we fixed the DOM of gelatin at 10%.^26^ Here, we investigated how varying the GEL_Tz and GEL_Nb ratio and polymer concentration affects hydrogel physicomechanical properties. A nonstoichiometric ratio (i.e., R05 vs R1) and a lower gelatin concentration (i.e., 8% vs 12%) led to lower rheological properties, higher swelling, and softer mechanical properties. These findings align with previous studies using different cross-linkers for gelatin hydrogels.^33−35^ Fine tuning the hydrogel physicomechanical properties enabled us to investigate their effect on the maturation of tooth buds.

Establishing epithelial-mesenchymal cell communication is critical for generating tooth organoids in hydrogels. In our experiments, we used dissociated mesenchymal and epithelial cells from mice embryos at the E14.5 stage (Figure S4D). At this stage, the odontogenic signal has already been transferred from epithelial cells to mesenchymal cells; thus the mesenchymal cells are expected to drive the induction and formation of the tooth organoids.^36^ In our previous study, we successfully demonstrated that dispersed dental pulp stem cells (DPSCs) exhibit a more elongated cell morphology within hydrogels characterized by lower mechanical properties.^26^ Additionally, our results in the whole tooth germ groups demonstrate how hydrogel physicomechanical properties influence tooth germ development. In native tissues, the ECM serves as a mechanical framework and triggers biological signaling in the cells. Several factors influence 3D cell behavior, including ECM stiffness.^37,38^ Here, we used hydrogels to mimic ECM and the stiffness of the hydrogel plays an important role in the organoid culture. It impacts the cellular communication dynamics, ultimately impacting the formation of tooth organoids.^39^

Our experimental design examines tooth organoid development at the cellular level in more detail while altering the 3D culture environment. The hydrogels used as scaffolds offer a tunable environment for tooth organoid formation by introducing appropriate polymer concentrations and Nb/Tz ratios. Fresh embryonic mouse cells were used exclusively, as both mesenchymal and epithelial cells lose odontogenic potential when cultured for extended periods of time.^40^ To potentially preserve these odontogenic signals, future studies could consider incorporating soluble factors within the hydrogel. Although no growth factors were included in this study, the adaptability of this hydrogel allows for the integration of specific molecules, thereby enhancing its functionality and versatility.

In this work, we evidenced the pivotal role that hydrogel properties play in shaping tooth organogenesis *in vitro* in artificial 3D matrixes. We designed hydrogels by changing their formulation and tuning their swelling, mechanical properties, and rheological properties, to show their involvement in determining the development of tooth germs at both tissue and organoid levels. Our findings highlight the essential role of biomaterials in facilitating tooth organogenesis for applications in developmental tissue engineering, regenerative dentistry, and *in vitro* tooth modeling.

## Supporting Information Available

Materials and methods, figures of variation of the hydrogel elastic modulus and hydrogel polymer density, staining images, percentage cell viability, H&E staining images, bright field images, and tables of bioorthogonally crosslinked hydrogel samples definitions and success rates of tooth organoid formation (PDF)
Tooth organoid in 3D culturing hydrogel (AVI)
Tooth organoid in 3D culturing hydrogel (AVI)

## Supplementary Material

mz4c00520_si_001.pdf

mz4c00520_si_002.avi

mz4c00520_si_003.avi

## Abbreviations

Tz: tetrazine
Nb: norbornene
GEL_Tz: gelatin decorated with tetrazine
GEL_Nb: gelatin decorated with norbornene
3D: three-dimensional
PGA: polyglycolide acid
PLLA: poly-l-lactide acid
PLGA: poly(lactic-*co*-glycolic acid)
GFP: green fluorescent protein
E14.5: embryonic day 14.5
ECM: extracellular matrix
DPSCs: dental pulp stem cells
DOM: degree of modification
GEL: gelatin hydrogels
