# Supplementary material for: Generating Tooth Organoids Using Defined Bioorthogonally Cross-Linked Hydrogels
Source: ACS Macro Lett. 2024 Nov 12;13(12):1620–6. doi: 10.1021/acsmacrolett.4c00520 (PMC11656705; doi:10.1021/acsmacrolett.4c00520)
Supplement: Supplementary file 1 — mz4c00520_si_001.pdf [file mz4c00520_si_001.pdf]

*Supporting information for*

**Generating Tooth Organoids using Defined Bioorthogonally Crosslinked Hydrogels**


Xuechen Zhang[a,‡], Nicola Contessi Negrini[b,c,‡], Rita Correia[b,c], Paul T. Sharpe[a], Adam D. Celiz[b,c,*], Ana Angelova Volponi[a,*]

[a] Centre for Craniofacial and Regenerative Biology, Faculty of Dentistry, Oral & Craniofacial Sciences, King's College London, Guy's Hospital, SE1 9RT London, UK

[b] Department of Bioengineering, Imperial College London, W12 0BZ, London, UK.

[c] The Francis Crick Institute, NW1 1AT, London, UK.

‡ These authors contributed equally.

*** Corresponding Authors:**

Ana Angelova Volponi: ana.angelova@kcl.ac.uk

Adam D. Celiz: a.celiz@imperial.ac.uk

## MATERIALS AND METHODS

### Materials

Conventional wild-type mice (CD1) and genetically modified mice expressing green fluorescent protein (GFP) were used in this study. Origin of GFP mice: male homozygous B6.129P2(Cg)-Cx3cr1tm1Litt/J mice[1] were purchased from the Jackson laboratory (Strain No. 005582) and crossed with CD-1 females to obtain offspring. All animal procedures conformed to the Animal Research: Reporting of In Vivo Experiments (ARRIVE) guidelines and in accordance with UK Home Office regulations.

Materials were purchased from Merck unless specified: gelatin (X-Pure low-endotoxin type B, gel strength 240−270 g, Rousselot Biomedical), tetrazine (methyltetrazine-amine, BroadPharm), norbornene (5-norbornene-2-methylamine, Nb, TCI Tokyo Chemical Industry), MES buffer (2-(N-morpholino)ethanesulfonic acid), deuterium oxide 3-(trimethylsilyl)propionic-2,2,3,3-d4 acid 0.05 wt% (TMSP), NHS (N-hydroxysuccinimide), EDC (N-(3-(dimethylamino)-propyl)-N-ethylcarbodiimide hydrochloride (Apollo Scientific), PBS (Dulbecco phosphate buffered saline), PDMS (polydimethylsiloxane, SYLGARD 184), Dispase II (Roche), Collagenase A (Roche), cell culture insert (0.4 µm; Falcon™), alpha-MEM (Gibco™), Penicillin-Streptomycin (Gibco™), FBS (Fetal Bovine Serum, Gibco™), PFA (Paraformaldehyde), mounting medium with DAPI (H-1200; VECTASHIELD).

### Hydrogel fabrication and characterization

Bioorthogonally crosslinked click hydrogels were prepared as previously described.[2] Briefly, gelatin hydrogel precursors were synthesized by decorating gelatin with tetrazine (GEL_Tz) or with norbornene (GEL_Nb). Gelatin was dissolved in MES buffer; Tz or Nb, EDC, and NHS were added at fixed molar ratios to achieve a total degree of modification of gelatin carboxylic groups of 10%.[3] After 4h under stirring at 37 °C, the gelatin solution was diluted 1:1 with distilled water, dialyzed for 4 days (MWCO 3.5 kDa), sterile filtered, and freeze-dried.

The Degree of Modification (DOM) of the functionalized polymers was determined using proton nuclear magnetic spectroscopy $^{1}$H NMR spectrometry (BrukerAvance 500 MHz spectrometer) and analyzed by MestReNova software. The DOM was calculated as Equation 1:[4]

$$\mathrm{DOM} = \frac{\int \mathrm{molecule}}{\int \mathrm{TMSP}} \times \frac{9\mathrm{H}}{2\mathrm{H}} \times \frac{\mathrm{n(TMSP)[mmol]}}{\mathrm{m(gelatin)(g)}} \qquad \text{[Equation 1]}$$

Where ∫TMPS is the internal reference signal (0 ppm/integrating for 9 protons); ∫molecule is the signal detected for Tz (8.5−8 ppm/integrating for 2 protons) in Tz-derivatives or the signal detected for Nb (6.3−5.9 ppm/integrating for 2 protons) in Nb-derivatives; n(TMPS) and m(gelatin) are the moles of TMPS and the mass of gelatin.

Gelatin hydrogels (GEL) were prepared by dissolving the gelatin hydrogel precursors (GEL_Tz and GEL_Nb, separately) in culture medium at 37 °C. The precursors were mixed, casted in PDMS molds, and allowed to crosslink at 37 °C for 2 h in humidified incubator. Three hydrogel

samples were prepared to investigate the effect of the polymer concentration and GEL_Tz/GEL_Nb ratio on the physico-mechanical hydrogel properties and biological response (Figure 2A). Gelatin hydrogel precursors was dissolved at either 8% w/V or 12% w/V (GEL_8% and GEL_12%, respectively); GEL_Tz and GEL_Nb were mixed at 1:1 and 0.5:1 volumetric ratio (GEL_R1 and GEL_R05, respectively). Sample names and formulations are summarized in Table 1.

The rheological properties of crosslinked hydrogels were tested (Netzsch Kinexus Ultra+) by shear strain tests to identify the linear viscoelastic region (parallel plates, diameter Ø = 25 mm, T = 37 °C, 1 Hz, 0.1-100% shear strain; n = 3), and by frequency sweep tests (parallel plate Ø = 25 mm, T = 37 °C, 1%, 0.1-100 Hz; n = 3). The hydrogel mesh size ξ was estimated following Equation 2:[5]

$$\xi = \sqrt[3]{\frac{6M_C}{\pi c N_{av}}} \qquad \text{[Equation 2]}$$

Where Mc is the average molecular weight between crosslinks, c is the concentration, and NAv is Avogadro's number.

The hydrogel swelling was investigated by immersing anhydrous samples in complete alpha-MEM (alpha-MEM with 10% FBS and 1% Penicillin-Streptomycin) at 37 °C to mimic *in vitro* culture conditions (n = 4). The percentage weight variation Δw [%] was calculated following Equation 3:

$$\Delta w[\%] = \frac{w_t - w_0}{w_0} \times 100 \qquad \text{[Equation 3]}$$

Where $w_t$ is the weight of swollen hydrogels at the time t, and $w_0$ is the initial dry weight. The hydrogel polymer density was calculated for freshly prepared hydrogels and hydrogels swollen for 8 days as the ratio between the polymer mass (i.e., freeze-dried samples) to the volume of the hydrated hydrogel (i.e., samples prior to freeze drying).

The mechanical properties of hydrogels (n = 3) were tested by compression tests (ElectroForce 5500), via one hysteresis cycle by loading at 5% min-1 (0.002 N preload) and subsequent unloading at 5% min-1, up to 30% strain. The Elastic Modulus (E) was calculated as the slope of the linear interpolation in the 0-5% strain loading curve.[6] The Elastic Modulus was evaluated on freshly prepared hydrogels (day 0) and on hydrogels during swelling (day 3 and day 8) to evaluate variation in *in vitro* culture-like conditions. Indentation tests (Biomomentum Mach-1) were performed to measure the instantaneous modulus of the swollen gels (n = 3), using a 5 mm diameter spherical indenter, displacement velocity 0.1 mm s-1, up to 20% of the sample thickness. The instantaneous modulus was calculated using the Hayes Model.[7]

The hydrogel cytocompatibility was evaluated using human dental pulp stem cells (hDPSC) as cell model. hDPSC were incorporated in the hydrogel precursors ($1\times10^6$ cell $mL^{-1}$); the precursors were mixed and crosslinked to obtain cell-laden hydrogels. After 1 day of culture, samples (n = 3) were washed with PBS and incubated with live/dead staining solution (calcein-AM 1uM, propidium iodine 5uM) for 45 min at 37 °C. Samples were washed three times in PBS and imaged (n = 3 images per sample) via confocal microscope (SP8 confocal Microscope,

Leica) to visualize and count viable (green) and dead (red) cells. The percentage of viable cells was calculated as ratio of the number of viable cells to the total number of cells.

**Embryonic epithelial and mesenchymal cells isolation**

The bilateral molar tooth germs were dissected from mouse embryos at embryonic day 14.5 (E14.5) of two types of mice with sterile needles (Figure 1 main text). The tooth germs were treated with Dispase II and incubated at 37 °C for 15 min. The epithelium from CD1 mice tooth germs and mesenchyme from GFP mice tooth germs were mechanically separated using tiny needles after being cleaned in PBS. Epithelium and mesenchyme were collected separately and transferred to 1 mg/ml Collagenase A to dissociate and ascertain the cell number. Mesenchymal and epithelial cells were sourced from E14.5 GFP and CD1 mouse embryo tooth germs, respectively and used in the experiments, where cells were easy to track and identify, showing *in vitro* bioengineered tooth organoids.

**Recombining mesenchymal and epithelial cells**

The recombination method of the cells was previously described (Figure 1, main text).[8,9] Equal number ($2 \times 10^5$) of mesenchymal and epithelial cells were centrifuged in a small tube to get cell pellets. The pellet was then injected into a hydrogel precursor (GEL_8%_R05, n = 13; GEL_8%_R1, n = 9; GEL_12%_R05, n = 6), and the cell-laden hydrogel was allowed to crosslink on a cell culture insert membrane (Day 0). Whole tooth germs were injected into separate hydrogels as control (n = 6). Complete alpha-MEM was added in the culture dish and cell-laden hydrogels were cultured *in vitro* for 8 days before characterization (Day 8).

**Histology and fluorescence imaging**

The samples were fixed using 4% PFA and subsequently washed with PBS. Paraffin and frozen section were used. For paraffin sections, after being dehydrated with ethanol solutions of increasing concentration, they were embedded in paraffin. For frozen section, the samples were embedded in OCT directly. A series of 7 μm thick sections were prepared and stained with hematoxylin and eosin (H&E). For fluorescence imaging, frozen section slides were mounted via mounting medium with DAPI and sealed with coverslips. Images were taken by an optical (NIKON ECLIPSE Ci-L) and a confocal (ZEISS LSM 980) microscope.

**Statistical analysis**

Data are represented as mean ± standard deviation. Differences between data groups were investigated by one-way ANOVA, with Tukey's multiple comparison (Prism-GraphPad software).

**SUPPLEMENTARY DATA**

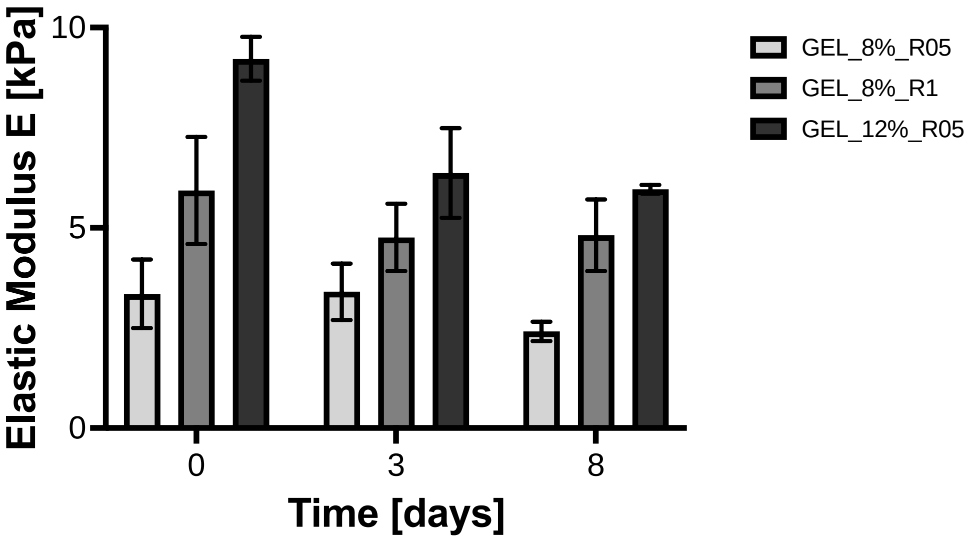


**Figure S1.** Variation of the hydrogel Elastic Modulus (E) during swelling in *in vitro* culture-like conditions (n = 3).

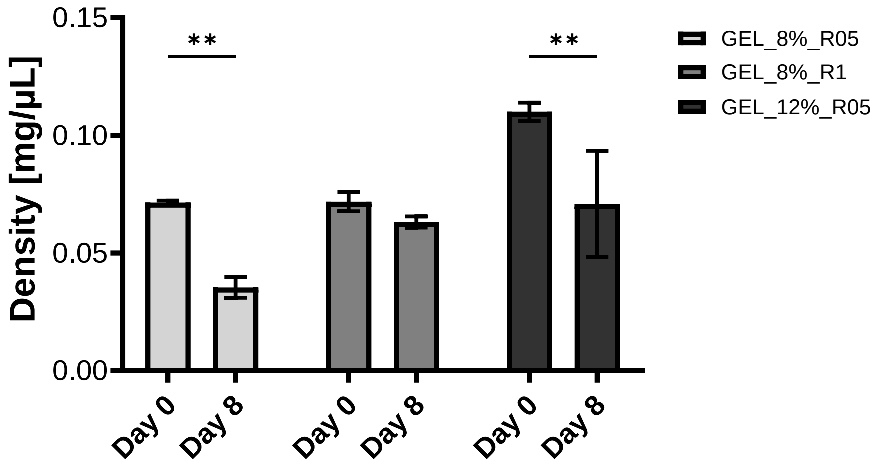


**Figure S2.** Variation of the hydrogel polymer density (n = 3) from freshly prepared hydrogels (day 0) to swollen hydrogels (day 8); ** $p < 0.01$.

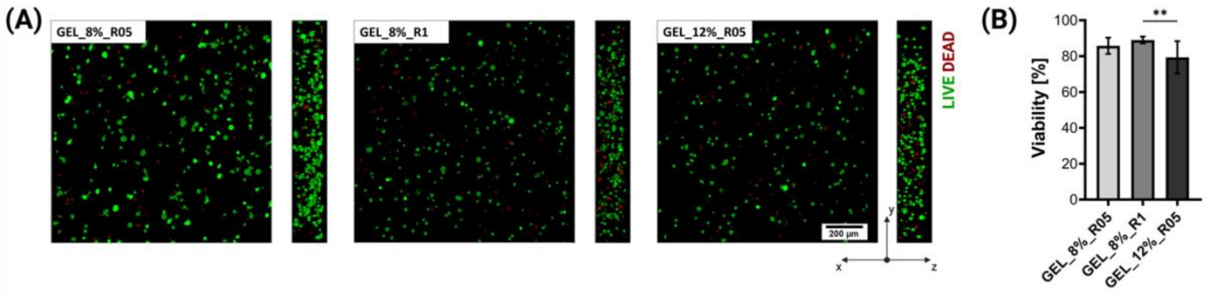


**Figure S3.** Human dental pulp stem cell-laden bioorthogonally crosslinked hydrogels. **(A)** Representative live (green)/dead (red) staining of hDPSC embedded in the 3D hydrogels (scale bar: 200 μm) and **(B)** percentage cell viability; ** $p < 0.01$. Created with Biorender.com.

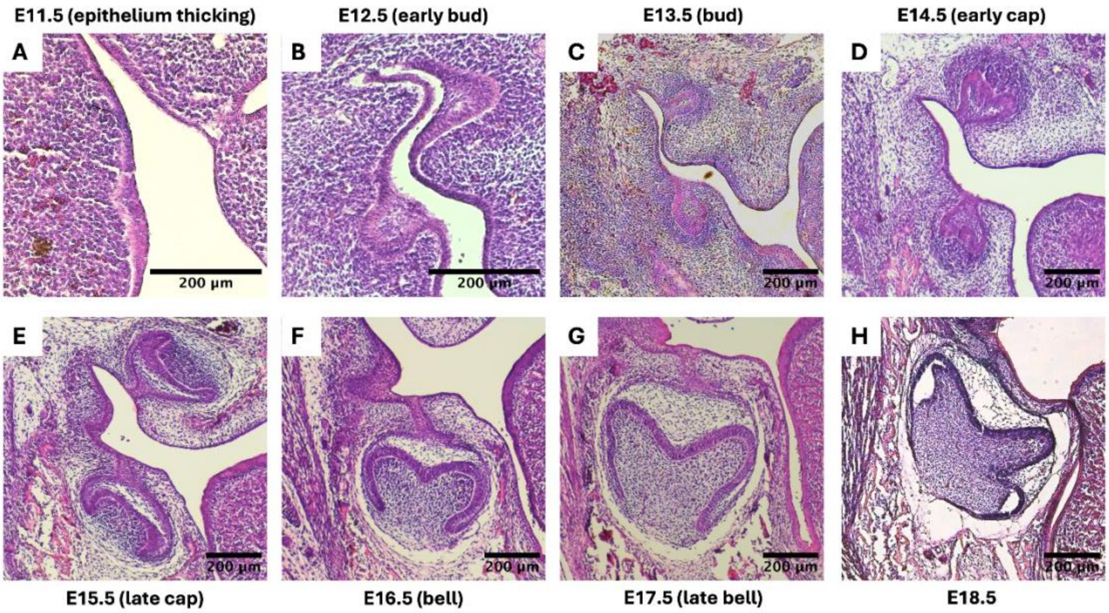


**Figure S4.** Representative H&E staining histological sections of molar tooth development in the mouse embryos at different stages from embryonic day E11.5 to embryonic day E18.5 (scale bar: 200 µm).

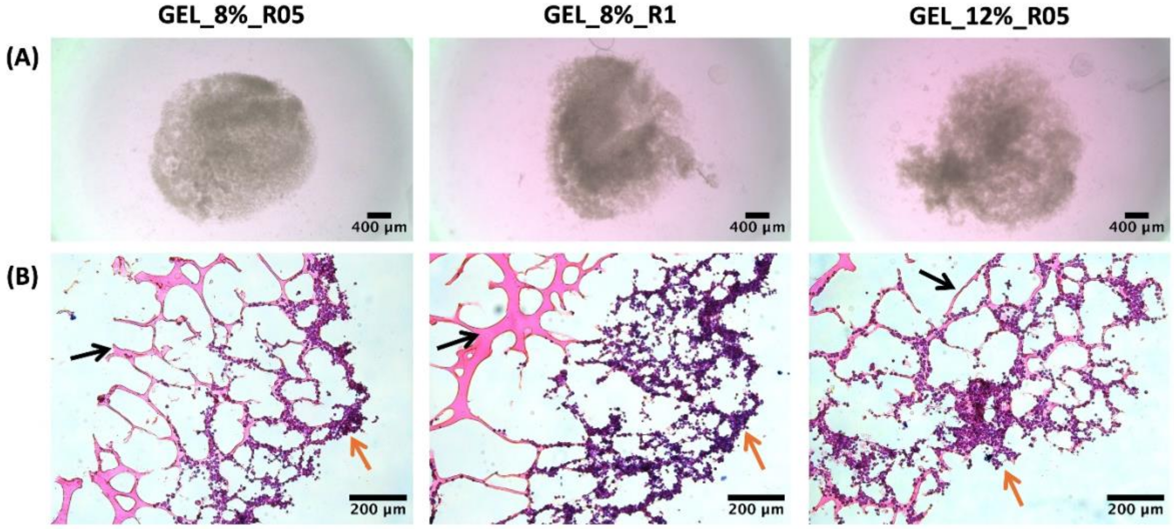


**Figure S5.** Representative (A) bright field images (scale bar: 400 μm) and (B) H&E staining histological sections (scale bar: 200 μm) of epithelial-mesenchymal cell pellets immediately after their encapsulation in bioorthogonally crosslinked gelatin hydrogels (GEL_8%_R05, GEL_8%_R1, and GEL_12%_R05; day 0). Black arrows: hydrogels; orange arrows: cells.

**Table S1.** Defined bioorthogonally crosslinked hydrogel samples prepared by varying the gelatin concentration (8 and 12% w/V) and the Gel_Tz:Gel_Nb precursor volumetric ratio (0.5 and 1).

| **Hydrogel sample** | **Gelatin concentration [% w/V]** | **Precursor volumetric ratio (GEL_Tz:GEL_Nb)** |
|---|---|---|
| **GEL_8%_R05** | 8 | 0.5 |
| **GEL_8%_R1** | 8 | 1 |
| **GEL_12%_R05** | 12 | 0.5 |

**Table S2.** Success rate of tooth organoid formation in different hydrogels.

| **Hydrogel sample** | **Number of experiments** | **Number of tooth organoids formed** | **Tooth organoid success rate** |
|---|---|---|---|
| **GEL_8%_R05** | 13 | 13 | 100% |
| **GEL_8%_R1** | 9 | 1 | 11% |
| **GEL_12%_R05** | 6 | 0 | 0% |

## REFERENCES


(1) Jung, S.; Aliberti, J.; Graemmel, P.; Sunshine, M. J.; Kreutzberg, G. W.; Sher, A.; Littman, D. R. Analysis of Fractalkine Receptor CX<sub>3</sub>CR1 Function by Targeted Deletion and Green Fluorescent Protein Reporter Gene Insertion. Molecular and Cellular Biology 2000, 20 (11), 4106-4114. DOI: 10.1128/mcb.20.11.4106-4114.2000 (acccessed 2024-07-23T11:55:09).

(2) Koshy, S. T.; Desai, R. M.; Joly, P.; Li, J.; Bagrodia, R. K.; Lewin, S. A.; Joshi, N. S.; Mooney, D. J. Click-crosslinked injectable gelatin hydrogels. Advanced healthcare materials 2016, 5 (5), 541.

(3) Contessi Negrini, N.; Angelova Volponi, A.; Sharpe, P. T.; Celiz, A. D. Tunable cross-linking and adhesion of gelatin hydrogels via bioorthogonal click chemistry. ACS Biomaterials Science & Engineering 2021, 7 (9), 4330-4346.

(4) Claaßen, C.; Claaßen, M. H.; Truffault, V.; Sewald, L.; Tovar, G. n. E.; Borchers, K.; Southan, A. Quantification of substitution of gelatin methacryloyl: best practice and current pitfalls. Biomacromolecules 2018, 19 (1), 42-52.

(5) Karvinen, J.; Ihalainen, T. O.; Calejo, M. T.; Jönkkäri, I.; Kellomäki, M. Characterization of the microstructure of hydrazone crosslinked polysaccharide-based hydrogels through rheological and diffusion studies. Materials Science and Engineering: C 2019, 94, 1056-1066.

(6) Negrini, N. C.; Bonnetier, M.; Giatsidis, G.; Orgill, D. P.; Farè, S.; Marelli, B. Tissue-mimicking gelatin scaffolds by alginate sacrificial templates for adipose tissue engineering. Acta Biomaterialia 2019, 87, 61-75.

(7) Hayes, W.; Keer, L. M.; Herrmann, G.; Mockros, L. A mathematical analysis for indentation tests of articular cartilage. Journal of biomechanics 1972, 5 (5), 541-551.

(8) Angelova Volponi, A.; Kawasaki, M.; Sharpe, P. Adult human gingival epithelial cells as a source for whole-tooth bioengineering. Journal of Dental Research 2013, 92 (4), 329-334.

(9) Yang, L.; Angelova Volponi, A.; Pang, Y.; Sharpe, P. Mesenchymal cell community effect in whole tooth bioengineering. Journal of Dental Research 2017, 96 (2), 186-191.
